# Bone Marrow-Derived Stem Cells: a Mixed Blessing in the Multifaceted World of Diabetic Complications

**DOI:** 10.1007/s11892-016-0730-x

**Published:** 2016-03-30

**Authors:** Giuseppe Mangialardi, Paolo Madeddu

**Affiliations:** Bristol Heart Institute, University of Bristol, Level 7, Bristol Royal Infirmary, Upper Maudlin Street, Bristol, BS28HW UK

**Keywords:** Diabetes, Diabetic complications, Bone marrow, Stem cells, Microangiopathy, Cell regenerative therapy

## Abstract

Diabetes is one of the main economic burdens in health care, which threatens to worsen dramatically if prevalence forecasts are correct. What makes diabetes harmful is the multi-organ distribution of its microvascular and macrovascular complications. Regenerative medicine with cellular therapy could be the dam against life-threatening or life-altering complications. Bone marrow-derived stem cells are putative candidates to achieve this goal. Unfortunately, the bone marrow itself is affected by diabetes, as it can develop a microangiopathy and neuropathy similar to other body tissues. Neuropathy leads to impaired stem cell mobilization from marrow, the so-called mobilopathy. Here, we review the role of bone marrow-derived stem cells in diabetes: how they are affected by compromised bone marrow integrity, how they contribute to other diabetic complications, and how they can be used as a treatment for these. Eventually, we suggest new tactics to optimize stem cell therapy.

## Introduction

Diabetes mellitus (DM) is a family of metabolic disorders characterized by high blood glucose levels. Worldwide, there are about 387 million people affected by DM, with a prevalence in North America of 11.4 % (39 million) and in Europe of 7.9 % (52 million) (http://www.idf.org/sites/default/files/DA-regional-factsheets-2014_FINAL.pdf). Estimation for 2035 foresees 592 million patients with diabetes on the planet. It is not surprising that DM is one of the heaviest economic health burdens, with an annual global cost of 548 million USD raising to 627 million in 2035 (http://www.idf.org/sites/default/files/DA-regional-factsheets-2014_FINAL.pdf).

There are several types of DM. Type 1 diabetes (T1DM) is an autoimmune disease characterized by β-cell destruction in the pancreatic islets resulting in the complete loss of insulin secretion. Type 2 diabetes (T2DM), the prevalent form of DM, is a multifactorial disease characterized by insulin resistance. Gestational diabetes (GDM) occurs in pregnant women without previous history of DM. Chronic hyperglycemia causes systemic complications, with cardiovascular complications being particularly frequent and worrisome [1]. Diabetic macrovascular disease manifests as coronary artery disease (CAD), myocardial infarction (MI), peripheral artery disease (PAD), and stroke. Microvascular disease results from damage to the small vessels and can aggravate the outcome of patients with occlusive macrovascular disease. Compared to hospital costs of people without complications, hospital costs of those with microvascular complications are doubled, those of people with macrovascular complications are tripled, and those of people with both micro- and macrovascular complications are six times as great. Lifestyle interventions, such as changes in diet and exercise, and medications are essential in reducing the risk for development of complications. Thanks to improved management, the prevalence of diabetic complications is constantly decreasing, but these improved clinical outcomes came with a cost of £708 million per year in the UK, corresponding to 7 % of the UK health-care prescribing budget [2]. Nevertheless, once cardiovascular complications occur, these are more difficult to treat in patients with DM as compared with patients without DM. This difference has been explained with the fact that healing and regenerative mechanisms are remarkably altered in patients with DM.

The current care of cardiovascular complications comprises pharmacotherapy and revascularization. However, medical treatment can be ineffective as in the case of refractory angina, which is prevalent in patients with DM. Additionally, in many patients with DM, revascularization cannot be applied due to multiple and distal occlusions, or it fails because of restenosis. But the most important limitation of medical and interventional treatments is that, even when successful, they do not replace cells irreversibly damaged by ischemia. Therefore, intense research is focused on regenerative medicine as a novel option for the treatment of cardiovascular complications. This approach consists of augmenting endogenous mechanisms of repair by supplements of regenerative cells, for instance through transplantation of stem cells/progenitor cells into the infarcted myocardium. Alternatively, cytokines and growth factors could be delivered to boost the liberation of progenitor cells from the bone marrow (BM) or the expansion of resident progenitor cells in the heart and other ischemic tissues. To date, a few thousand individuals worldwide have received stem cells—mainly BM cells—in cardiovascular clinical trials [3].

In this review, we provide a concise report of the latest findings about cell therapy for treatment of diabetic complications, with particular focus on approaches based on BM-derived cells. We also discuss the pros and cons of BM cell therapy, especially in light of new discoveries that common complications of DM, namely microangiopathy and neuropathy, affect also the BM and may jeopardize stem cell mobilization following an ischemic event or stimulation by cytokines. Finally, we propose some possible solutions to improve current cell therapy approaches.

## Bone Marrow-Derived Stem Cells and Their Niches

BM is the only reservoir of human pluripotent stem cells (HPSCs) in the adult human body, providing a specific microenvironment to support stem cell maintenance and/or expansion. Here, we provide a brief overview of the different cell populations of interest and their organization in the BM niche.

### Hematopoietic Stem Cells

Hematopoietic stem cells (HSCs) constitute a very heterogeneous population, which can give rise to any blood cell. They are defined as proper stem cells because of their self-renewal capacity and multipotency [4]. BM transplantation assays in animal models helped to establish a possible hierarchical structure. Accordingly, based on repopulating capacity upon transplantation into irradiated animals, two different populations have been identified and defined as short-term HSCs (ST-HSC) and long-term HSCs (LT-HSCs) [5]. In humans, the surface marker CD34, a member of a family of single-pass transmembrane glycoproteins, is considered the main defining marker for HSCs and the marker of choice to select populations for clinical cell therapy trials. Depleting CD34+ cells for Lin antigen enables their enrichment into a more primitive population with hematopoietic function. Further enrichment could be achieved by selecting the latter population for Thy1, a primitive stem cell marker, and depleting for CD38 and CD45RA markers (multipotent progenitors), with additional positivity for CD49f defining proper LT-HSCs [6]. In the mouse, LT-HSCs are identified as CD34−/c-Kit+/Sca-1+/Lineage− (CD34−/KSL) cells. However, CD34−/KSL cells represent a heterogeneous population containing sub-fractions with diverse regenerative capacity. Further characterization by use of the signaling lymphocyte activation molecule (SLAM) markers CD48 and CD150 allows the identification of a fraction of CD34−/KSL cells endowed with high self-renewal potential and repopulating capacity [4]. The seminal concept of BM HSCs directly participating in de novo post-natal vasculogenesis, proposed some years ago by Asahara et al. [7], has been revised in light of the novel indication that paracrine mechanisms, rather than the conversion of one cell type to another, control the interrelation between hematopoietic and vascular cells [8].

### Mesenchymal Stem Cells

Mesenchymal stem cells (MSCs) are a heterogeneous multipotent stromal cell population well represented in BM as well as in several other adult tissues. They are defined as plastic-adherent, fibroblast-like cells endowed with high proliferative activity and capacity to differentiate into a variety of cell types, including osteoblasts (bone cells), chondrocytes (cartilage cells), myocytes (muscle cells), and adipocytes (fat cells) [9]. They typically express the mesenchymal markers CD44, CD90, CD49, CD54, CD105, and CD73, but are negative for both hematopoietic markers, such as CD45, CD14, and CD11b, and endothelial markers, such as CD144, von Willebrand factor (vWf), and VCAM-1 [9]. MSCs have been shown to possess special plasticity. In fact, under appropriate experimental conditions, they can also differentiate into non-mesenchymal cells—for instance, neural cells and endothelial cells [10]. In addition, MSCs exert pro-angiogenic and immunomodulatory activities, through the secretion of different cytokines/growth factors [11], altogether making MSCs a popular choice for regenerative medicine applications.

### Endothelial Progenitor Cells

Endothelial progenitor cells (EPCs) are angiogenic cells characterized for the first time by Ishikawa and Asahara in 1997 [12]. Since their discovery, many definitions have been proposed, creating a maelstrom of overlapping nomenclatures. Scientific consensus agreed on defining EPCs as a subset of myeloid/monocyte cells which can acquire endothelial markers, such as CD31, KDR, or vWf, together with lectin-binding ability in in vitro culture conditions [13]. They have to be considered a different population from endothelial colony-forming cells (ECFCs), a non-marrow-derived population, and more resembling endothelial cells likely originated from vessel-associated progenitor cells [14]. EPCs exert their pro-angiogenic potential in a paracrine fashion rather than differentiating in vascular cytotype [15]. EPCs have been extensively studied in animal models and clinical trials thanks to their supportive role in angiogenesis.

### Localization of Stem Cells in the BM Niche

The BM is found within the cavities of long bones. The trabecular bone structure endowed with spare adipocytes provides a supportive meshwork for niches. Two different types of niches have been identified so far. The osteoblastic niche localized near the endosteal part of the bone provides a shelter for most primitive HSCs. A mixture of different cells contributes to the maintenance of HSC homeostasis. In particular, osteoblasts and macrophages play a pivotal role in regulating the endosteal niche milieu. Osteoblasts regulate the niche through Wnt signaling, and their depletion leads to niche pauperization [16]. A particular subset of macrophages, expressing CD169, could regulate the abundance of osteoblasts and also regulate the retention-mobilization of HSCs [17]. The vascular niche is organized around sinusoidal vessels and harbors more committed HSCs. In this niche, endothelial cells and perivascular cells are the main regulators. Endothelial cells mediate maintenance of HSC homeostasis through production of Akt-related angiocrine factors, such as angiopoietin-1, delta-like 1, insulin-like growth factor-binding protein 2, and epidermal growth factor [18]. Perivascular cells positive for CD146, platelet growth factor receptor α, and nestin could be recognized around the sinusoids. They express two master regulators of HSC homeostasis: the chemokine (C-X-C motif) ligand 12 (CXCL-12) and stem cell factor (SCF) [19]. It has also been observed that perivascular cells exert their effects through cell-to-cell contacts via the Notch signaling pathway [19]. This spatial organization creates a gradient, in which HSCs could progress from the most primitive self-renewal state to lineage commitment, ready for egression into the bloodstream. Additionally, reactive oxygen species (ROS) distribute according to the BM perfusion gradient, with higher levels in the vascular niche and lower levels in the endosteal niche [20•]. This gradient contributes in maintaining the balance between stem cell self-renewal and differentiation across the niches [20•].

## The Upside: Evidence for BM-Derived Stem Cells Improving Diabetic Complications

### Diabetic Retinopathy

Diabetic retinopathy (DR) is the major cause of vision impairment in working-age adults in western countries [21]. The main accepted mechanism in DR development is related to the loss of pericytes. They provide a nourishing, anti-inflammatory, and stabilizing microenvironment for human retinal endothelial cells (HREC). In the early stage, DM impairs pericyte self-renewal and endothelial cell survival, resulting in a hypoxic retinal microenvironment [22]. This triggers the translocation of hypoxia-inducible factor-1 alpha (HIF-1a), which in turn leads to the upregulated expression of vascular endothelial growth factor A (VEGF-A). Persistent VEGF-A production stimulates retinal angiogenesis, fibroblast scar formation, and vascular leakage, resulting in proliferative diabetic retinopathy (PDR) [22].

Cell therapy has been proposed as a novel approach to treat diabetic PDR. In this context, MSCs emerged as one of the most promising candidates for several reasons. A large body of evidence points out an equivalence between MSCs and pericytes [23], making MSCs the ideal replacement for the latter. Moreover, BM-derived MSCs have the ability to partially differentiate into photoreceptors and retinal pigmented epithelium in vitro and in vivo [24]. This ability is important considered that neurodegenerative events, including glial cell reactivity, microglial activation, and neuronal apoptosis, take place at the early stage of DR [25]. First phase I/II clinical trials using adult MSCs have been carried out (https://www.clinicaltrials.gov, NCT01068561 and NCT01560715) in retinitis pigmentosa. In the RETICELL clinical study, Siqueira et al. demonstrated an improvement in patient quality of life, unfortunately only for a short time, as any beneficial effect had disappeared after 12 months [26]. Currently, only two clinical trials with intravitreal injection of adult MSCs in patients suffering from ischemic retinopathy and macular degeneration are ongoing (https://www.clinicaltrials.gov, NCT01518842 and NCT01518127).

Besides MSCs, CD34+ HSCs represent a valid alternative. In different animal models, intravitreally injected CD34+ cells showed, similarly to MSCs, the ability to rescue retinal vasculature and a neuronal protective effect [27]. More importantly, intravitreal injection of BM-derived CD34+ cells is safe and well tolerated on the long term [27]. Park et al. detected CD34+ cells after 8 months from injections, with no histological sign of intraocular tumor or abnormal tissue growth [28]. Those premises paved the way to the first clinical trial (https://www.clinicaltrials.gov, NCT01736059) in patients who are irreversibly blind from various retinal conditions, including DR. In vivo imaging showed changes suggestive of new cellular incorporation into the macula of the hereditary macular degeneration study [29]. Previously, Siqueira et al. treated patients with macular edema associated with macular ischemia, observing an improvement in patient visual performances [30].

### Diabetic Cardiomyopathy

Diabetic cardiomyopathy (DCM) was firstly described in 1972 by Rubler et al. who observed congestive heart failure in patients with no coronary artery disease (CAD) [31]. The definition of DCM has evolved through the years and to date comprises the presence of diabetic microangiopathy, myopathy, and autonomic neuropathy. Briefly, DCM is characterized by an increased left ventricular (LV) mass and wall thickness frequently associated with cardiomyocyte hypertrophy [31]. Similar to other diabetic complications, DCM likely originates from altered metabolism related to chronic hyperglycemia. Insulin resistance leads to a switch from glucose oxidation metabolism to free fatty acid oxidation which is more oxygen demanding. Those events trigger cardiac ischemia inducing the shift to anaerobic metabolism, which in turn produces more lactate and acid metabolites resulting in lipid accumulation and altered cardiomyocyte contraction and apoptosis [32]. Increased oxidative stress and advanced glycation end product (AGE) formation contribute to fibrosis and cell death, while the occurrence of endothelial dysfunction manifests as altered permeability and increased leukostasis, leading to myocardial edema and inflammation [32].

Cell therapy is expected to benefit DCM through regenerative, pro-angiogenic, anti-fibrotic, and anti-inflammatory mechanisms. MSCs showed the in vitro and in vivo ability to differentiate into cardiomyocytes [33]. In the seminal work by Nagaya et al., MSC therapy was able to significantly increase capillary density and to decrease the collagen volume fraction in the myocardium, resulting in decreased left ventricular end-diastolic pressure [33]. The protective effect exerted by MSCs is seemingly attributed to remarkable paracrine activity. For instance, in vivo studies showed that intra-cardiac injection of MSCs in a rat model of post-ischemic heart failure led to a significant decrease in ventricular fibrosis associated with improved cardiac parameters. The effect was mainly mediated by the inhibition of MMP-9, a mediator of apoptosis in DCM, and the promotion of MMP-2 activity, which inhibits collagen deposition [34]. Additionally, MSCs protect cardiomyocytes from apoptosis by upregulating 14-3-3 and p-Ask1 proteins [35]. Moreover, MSCs are able to decrease immune cell infiltration [36]. Cytokines and growth factors could be used to potentiate stem cell mobilization and homing to the myocardium. Improved mobilization with granulocyte colony-stimulating factor (G-CSF) ameliorates the rat model of CDM decreasing perivascular interstitial fibrosis [37]. Our recent study in BM cells from infarcted patients demonstrated that SDF-1 directed migration enriches for cells with increased healing activity [38]. Additionally, BM-derived EPCs not only promote neovascularization but also exert anti-fibrotic action in diabetic hearts by paracrine inhibition of miR-155 [39].

To date, BM cell therapy has been tested in several clinical trials on patients with acute or chronic heart failure, producing mixed results [40]. However, to the best of our knowledge, no clinical trial has been so far conducted with BM cells for treatment of DCM.

### Diabetic Nephropathy

Diabetic nephropathy is the leading cause of end-stage renal disease (ESRD) worldwide. Diabetic kidney disease is characterized by glomerular hypertrophy, basement membrane thickening, mesangial expansion, tubular atrophy, interstitial fibrosis, and arteriolar thickening. Those morphological changes are attributable to chronic hyperglycemia and enhanced oxidative stress. Podocyte dysfunction is key in these pathological process.

BM cell therapy has been tested in animal models of diabetic nephropathy with promising results. In 2006, Lee et al. reported that intracardial injection of MSCs in a T1DM mouse model improves metabolic control as well as glomerular morphology, mesangial thickening, and macrophage infiltration [41]. In a murine model of DM, Ezquer et al. obtained similar results, even if no restoration of normal glucose levels was observed. Interestingly, MSCs were detectable after 3 months of injection [42]. In a rat model of diabetic nephropathy, improved podocyte function was paralleled by decreased albuminuria [43]. Similar studies in a diabetic rat model provided mechanistic insight of autologous MSC transplant. The reno-protective mechanism of MSC therapy includes inhibition of oxidative stress, possibly through improved insulin sensitivity leading to better metabolic control [44]. Furthermore, rat bone marrow-derived MSCs decrease macrophage infiltration by downregulation of cytokines, like monocyte chemotactic protein 1 (MCP-1), interleukin 1β (IL-1β), interleukin 6 (IL-6), and tumor necrosis factor α (TNF-α) [45]. Additionally, VEGF and anti-apoptotic protein B cell lymphoma 2 (bcl2) are upregulated and transforming growth factor β (TGF-β) and pro-apoptotic factor Bax are downregulated [46]. Inhibition in the TGF-β signaling pathway is associated with decreased fibrosis, with this mechanism being likely mediated by secreted protein osteogenic protein-1 (OP-1) [47]. A study from Zhang et al. showed similar results in a db/db mouse model injected with EPCs [48]. Finally, recruitment of healing BM cells could occur in the beneficial effects of angiotensin receptor antagonists in preventing diabetic glomerulosclerosis [49].

### Diabetic Neuropathy

Diabetic neuropathy (DNP) is a common and disabling manifestation of DM and often overlaps with vascular complications. Sensory neuropathy is a typical form of peripheral neuropathy characterized by an altered perception of noxious stimuli. From a functional standpoint, it manifests as an inability of neurons to produce proper amounts of neuropeptides, like substance P (SP) and calcitonin gene-related peptide (CGRP), in response to tissue injury.

Pre-clinical cell therapy studies showed promising results for treatment of DNP. In a T1DM rat model, MSCs ameliorated hypoalgesia and improved nerve conduction velocity, sciatic nerve blood flow, and capillary number-to-muscle fiber ratio in soleus muscles [50]. MSCs showed the potential to support direct de novo regeneration of neuronal cells [51]. However, paracrine properties seem to be prevalent. Indeed, MSCs produce a list of anti-inflammatory cytokines, which moderate leukocyte recruitment, together with neurotrophic and angiogenic factors such as nerve growth factor (NGF), VEGF, and IGF-1 [52]. This dual action on nerves and vessels is also exerted by EPCs. In a T1DM mouse model, EPC administration increased VEGF, basic fibroblast growth factor (bFGF), and glioma-associated oncogene family zinc finger 1 (Gli 1) protein, which was associated with increased proliferation of Schwann and endothelial cells. Interestingly, engrafted EPCs tended to co-localize near the vasa nervorum [53]. Differently from other BM cell populations, freshly isolated BM-derived mononuclear cells (MNCs) produced prevalent improvements on nerves in a DNP rat model [54]. As reported for other complications, in vivo data support the possibility to explore endogenous cell mobilization as a therapeutic alternative to classical cell therapy. In a rodent model of DNP, intraperitoneal administration of G-CSF improved nerve function, preventing axonal atrophy and demyelination, mainly due to peri-neuronal recruitment of stem cells from BM. However, no fusion with recipient cells or proof of BM cell differentiation into neurons was observed, thus suggesting a paracrine action by recruited cells [55].

Currently, no data from clinical trials are available regarding BM cell therapy for treatment of DNP in patients.

### Macrovascular Complications

Critical limb ischemia (CLI), the end stage of peripheral artery disease (PAD), is caused by severe obstruction of blood flow resulting in unbearable pain, ulcers, and a high risk for amputation. Surgical bypass or percutaneous revascularization, the gold standard for the treatment of CLI, exerts only temporary symptomatic alleviation. Furthermore, around one third of patients with CLI cannot be revascularized because of multivascular disease or occlusions of small-caliber blood vessels [56]. Gene and cell therapies have been therefore proposed as a possible complement or alternative to interventional angioplasty.

Different pre-clinical and clinical trials have been carried out in the recent years, mainly using BM-derived MNCs or EPCs, with mixed results. A recent systematic review of clinical trials with intramuscular delivery of BM-derived MNCs in patients with CLI indicates that there is no evidence to support this therapeutic approach. Only in one study was a lower rate of amputation appreciated, pointing out that larger randomized controlled trials are needed in order to provide adequate statistical power [57]. On the other hand, another systematic review identified 13 studies with proper controlled groups using autologous transplantation of BM-derived stem cells. Taken together, these studies suggest BM cells were well tolerated and improved ischemic symptoms in patients with DM, prolonging the amputation-free survival period and promoting complete wound healing [58]. A recent double-blind, randomized, placebo-controlled, phase I/II study showed MSCs ameliorate the ankle brachial pressure index (ABPI) and ankle pressure in patients with established CLI [59]. Conversely, autologous supplementation of CD133+ cells through mobilization showed poor results due to senescence of the stem cell compartment [60].

BM-derived stem cells have been also thoroughly investigated in randomized clinical trials for stroke or acute myocardial infarction (AMI) treatment. Despite differences on the timing of cell transplantation after stroke onset, on the short- or long-term follow-up, and on cell-culture conditions, all studies have led to a significant functional improvement, without adverse effects [61]. However, the limited number of participants enrolled in those studies, associated with limited bioavailability of BM stem cells in persons with DM, demands further investigation to overcome these issues [61]. In AMI treatment, the majority of clinical trials used BM MNCs [62, 63]. Gathered results demonstrated BM MNCs to be safe on the long term, but modest hemodynamic improvements were appreciated [63]. Additionally, MSCs have been used in clinical AMI trials. Although their use is safe, no definitive results have been achieved yet [64].

## The Downside: Evidence for Impairment of BM-Derived Stem Cells Contributing to Diabetic Complications

The BM is emerging as just one additional tissue stricken by complications of DM. Initial data from pre-clinical and clinical studies indicated DM causes quantitative and qualitative impairments in circulating stem cells [65]. However, initial damage directly occurs at the source-tissue level, before cells are mobilized to the peripheral circulation, due to a remarkable remodeling of all the components of the marrow niche [66••]. These changes lead to the disruption of stem cell homeostasis, providing a further mechanism to explain and interpret peripheral complications.

### Bone Marrow Microangiopathy

The BM is perfused by a complex network of vessels, formed by penetrating arteries, distal arterioles, and sinusoidal capillaries. Sinusoidal capillaries form highly branched and irregular networks and are primarily found between hematopoietic cells within the marrow cavity. Arteries contain comparably few side branches and are mainly associated with bone-forming cells, i.e., osteoblasts. Our seminal work provided the first evidences that diabetic BM undergoes profound structural alterations [67]. Using a T1DM mouse model, we reported a severe microvascular rarefaction responsible for marrow hypoperfusion. Isolated endothelial cells from diabetic mice presented higher levels of ROS production and upregulation of the senescence marker β-galactosidase [67]. Endothelial dysfunction was confirmed in a subsequent study showing an increased vascular leakage of proteins and leukocytes, both hallmarks of vascular diabetic complications. This was associated with an increased activity of redox-sensitive RhoA GTPase and downstream kinase ROCK 1/2. Both RhoA silencing with a dominant negative form and ROCK inhibition rescued endothelial dysfunction, restoring Akt-dependent production of angiocrine factors, and vascular permeability [68•]. DM also affected the stem cell compartment as HSCs were mostly depleted in the extended, low-perfused part of the marrow [67]. Those findings were confirmed in a T2DM mouse model [69], as well as in people with T2DM [66••]. Histopathological measurements of human BM documented microvascular rarefaction at the sinusoid and arteriole levels, osteopenia, and increased adipogenesis [66••]. Molecular studies highlighted the downregulation of miRNA-155, a master regulator of HSC self-renewal, to be a key underpinning mechanism of diabetic CD34+ cell pauperization [66••]. The occurrence of remodeling of source tissue may represent a serious obstacle to efficient harvesting or mobilization in view of autologous transplantation of CD34+ cells. In fact, a recent study in participants with refractory angina has shown that the efficacy of this treatment is clearly dose dependent [70]. This concept is strengthened by the observation of a delayed repopulating capacity of diabetic BM CD34+ cells [71].

### BM Neuropathy and Mobilopathy

Another master regulator of stem cell homeostasis and trafficking is represented by the BM sensorial and autonomic innervation. HSC release into the bloodstream is regulated by a circadian cycle through the activity of noradrenergic fibers [72]. Sympathetic and nociceptive fibers stimulate stem cell release either directly or indirectly through modulation of stromal cells, i.e., perivascular cells and osteoblasts [73]. Katayama et al. recently showed that HSC mobilization by G-CSF was reduced in sympathectomized mice [74]. They also showed that G-CSF attenuates osteoblast function, via the sympathetic nervous system, thereby facilitating HSC detachment from the endosteal niche. In addition, human CD34+ cells express β2-adrenergic and nociceptive receptors that are upregulated by G-CSF [75]. These studies demonstrate that neurotransmitters serve as direct chemoattractants to HSCs under conditions where the mobilization of regenerative cells is required to promote healing of injured or ischemic peripheral tissues [71]. In fact, abrogation of nociceptive signaling by genetic or pharmacological manipulation results in delayed recovery from ischemia [73]. Likewise, the occurrence of autonomic neuropathy accounts for the known defective mobilization of HSCs in persons with DM [76, 77]. A clinical trial (https://www.clinicaltrials.gov, NCT01102699) demonstrated that participants with DM challenged with G-CSF have a reduced mobilization of HSCs, due to altered expression of dipeptidyl peptidase-4 (DPP4, also known as adenosine deaminase complexing protein 2 or CD26) [78, 79]. DPP-4’s natural substrate is the chemokine SDF-1α, a major regulator of the stem/progenitor cell trafficking between the BM and circulation. A meta-analysis review on clinical trials pointed out that G-CSF is the main affected mobilization signaling pathway in people with DM [80]. Several studies in animal models replicated the human findings and provided an insight for the underlying mechanisms [81]. In particular, Albiero et al. demonstrated that both experimental murine models of T1DM and T2DM develop BM autonomic neuropathy with an impaired mobilization of HSCs. This effect is mediated by upregulation of p66Shc and downregulation of Sirtuin 1 (Sirt1), together leading to elevated expression of the adhesion molecule CD62L (L-selectin). Knock-out of p66Shc, as well as overexpression of Sirt1, was able to restore proper stem cell trafficking [82•]. In our recent study, we demonstrated a rarefaction in nociceptive fibers in the BM of patients with T2DM [83••]. This was associated with a drastic reduction of NK1R, the receptor for nociceptive transmitter SP [83••]. In addition, following induction of limb ischemia, diabetic mice showed an incapacity to create a peripheral-to-BM SP gradient. As said before, this gradient drives the mobilization and homing of HSCs to the ischemic site [73]. Consequently, there was a reduction in the recruitment of NK1R+ HSCs to the ischemic limb muscles of diabetic mice, resulting in delayed recovery and depressed reparative angiogenesis [83••]. Collectively, these reports indicate that neuropathy conjointly with microangiopathy may adversely affect stem cell-based therapies in patients with DM.

### Adipogenesis and Bone Resorption

Along with microangiopathy and autonomic neuropathy, increased adipose tissue is a hallmark of BM remodeling in DM, both in animal models and humans [66••, 67]. These changes are likely due to enhanced adipogenesis from MSCs [84]. This is associated both in humans and animal models with a decrease of the ratio between trabecular bone mass and adipose tissue [66••, 67]. Increased deposition of adipose tissue, as well as a reduction in the number of osteoblasts, could be enlisted among the causes of stem cell pauperization observed in diabetic BM. In fact, adipocytes can disturb the cellular network inside the niches by releasing oxidative and inflammatory mediators [85], activating the AGE pathway [86], and modifying the metabolic milieu [87].

### BM-Derived Stem Cells Involved in Other Complications

In previous sections, we have illustrated how DM complications extend to the BM and impact on stem cells and their niche. The movement of damaged cells along with a spectrum of lineage-committed cells into the circulation may pave the way for the enhancement of peripheral complications. This assumption is supported by new experimental evidence showing that BM-derived cells can actively participate at the two different stages of DR. In the non-proliferative stage, BM-derived CD11b-positive cells contribute to capillary degeneration through leukostasis [88]. As demonstrated by Li et al., BM-mobilized monocytes can contribute to the generation of oxidative stress and vessel regression [89]. On the other hand, BM-derived EPCs play a pivotal role in the angiogenic stage of DR via the secretion of growth factors and chemokines [90]. Studies investigating the correlation of EPC numbers and their contribution to DR in humans produced mixed results. For instance, CD34+ cells were found either increased, decreased, or unchanged in T2DM-associated DR [91, 92], whereas in T1DM, EPC numbers correlate well with the proliferative grade of the disease [93, 94]. With regard to diabetic nephropathy, clinical studies pointed out a correlation between microalbuminuria and circulating CD34+ cells [95, 96]. Only pre-clinical data are available regarding diabetic neuropathy. In an elegant study using a T1DM animal model, Terashima et al. showed that BM cells are indeed involved in the pathobiology of diabetic neuropathy, being able to fuse with neurons in the dorsal root ganglia [97]. In a following study, they found similar results in diabetic animals, demonstrating these fusion events occur between neuronal cells and a specific subset of HSCs expressing insulin and TNF-α [98•]. The link between BM pathology and vasculopathy seemingly involves the spleen as a mandatory site of cell commitment to an inflammatory phenotype. It is well known that patients with acute coronary syndromes have a high risk of recurrent events. This was ascribed to mobilization of myeloid cells that have disruptive activity of atherosclerotic plaques. These hematopoietic stem and progenitor cells are liberated from BM niches via sympathetic nervous system signaling. They then colonize the spleen, yielding a sustained boost in monocyte production [99].

## Restore BM Functionality to Improve Cell Therapy of Diabetic Complications

**Fig. 1:**
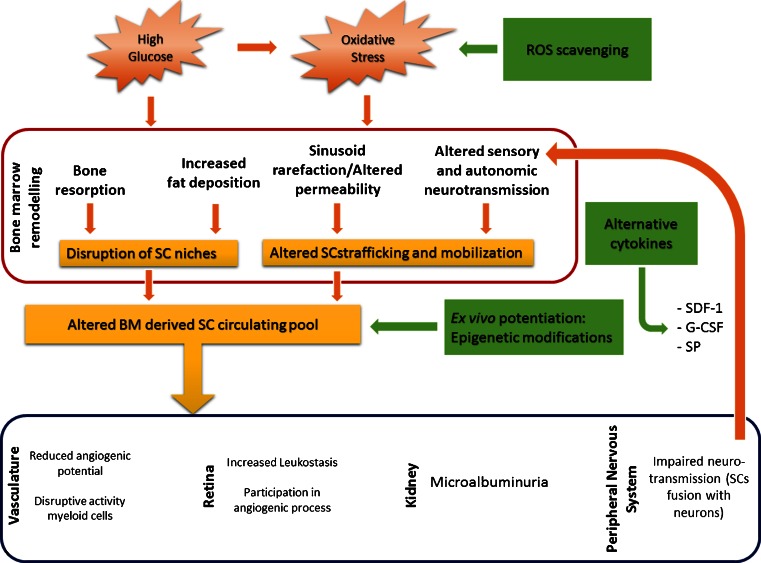
Mechanisms responsible for bone marrow remodeling under chronic hyperglycemia and oxidative stress and potential therapeutic applications. The *orange box* represents early pathobiological events which trigger later ones, shown in the *yellow boxes*. Possible therapeutic applications are shown in the *green boxes*

Data reported in previous sections suggest that DM poses serious challenges to current stem cell therapies. The obvious approach to interrupt the vicious cycle between peripheral and BM damage is to reinforce classical interventions for achievement of proper and consistent metabolic control (Fig. 1). Well-designed clinical trials similar to the UK Prospective Diabetes Study should be performed to establish the association between stable normalization of hyperglycemia and improvement of BM pathology. Performing such a study will be complicated by the need of an invasive procedure to collect BM material. Alternatively, analyses of circulating cells could be used as a surrogate endpoint. The use of ROS scavengers could also provide opportunities. Studies in experimental models indicate that abrogation of p66Shc results in protection from angiotensin II-induced cardiac damage [100], improvement of reparative angiogenesis and reduction of myocyte apoptosis following induction of limb ischemia, and preservation of satellite cell proliferation and differentiation under in vitro conditions of high oxidative stress [101, 102]. Blockade of this pathway by specific inhibitors of protein kinase C β (PKCβ), the enzyme responsible for p66Shc phosphorylation/activation, was demonstrated to ameliorate the vascular dysfunction in diabetic animals, providing a new strategy for the treatment of diabetic vascular complications [103, 104]. Indeed, the PKCβ inhibitor LY333531, also known as ruboxistaurin, is under advanced investigation in clinical trials to treat DR. It would be interesting to investigate if PKCβ inhibitors are useful in preventing or rescuing BM stem cell availability and functionality in DM.

Thiamine pyrophosphate is the cofactor for transketolase, the rate-limiting enzyme that shunts glyceraldehyde 3-phosphate and fructose 6-phosphate from glycolysis into the non-oxidative branch of the pentose phosphate pathway. Thiamine deficiency has been reported in diabetes, and correction of the defect by supplementation of thiamine or its derivative, benfotiamine, was shown to protect against diabetic nephropathy and retinal microangiopathy [105]. We have also shown that benfotiamine supplementation prevented microangiopathy and hypoperfusion of diabetic BM. Furthermore, benfotiamine reduced oxidative stress and oxidative damage of DNA in BM cells. Importantly, these effects were associated with prevention of HSC depletion, both in terms of absolute number and relative proportion to total BM cells, and inhibition of apoptosis [67]. Clinical studies using benfotiamine are therefore eagerly waited. Moreover, similar strategies could be useful to counteract neuropathy and related insensitivity to chemokine stimulation, e.g., mobilopathy [82•].

Mobilopathy could also benefit by the use of alternative cytokines different from G-CSF. In a retrospective study, Fadini et al. observed that inability of G-CSF to mobilize stem cells in patients with DM was abrogated when administrated in conjunction with pleraxifor, an antagonist of the SDF-1/CXCR4 axis [106•]. Moreover, our study on nociceptive signaling paves the way for the application of modulators of pain as a novel strategy to help proper mobilization [83••].

Alternatively, specific treatment on ex vivo isolated cells could help to restore their full regenerative potential. As discussed earlier, BM-derived cells can be isolated and expanded ex vivo for autologous or allogeneic transplantation. Gene therapy could be a viable approach in order to rescue stem cell functionality. For instance, the microRNA-155 downregulation in HSCs represents a candidate target to maintain the pool of original stem cells in diabetic BM [66••]. Conversely, silencing of microRNA-15a and microRNA-16 could ameliorate EPC angiogenic potential [107]. A less problematic approach would be the pre-conditioning of BM-derived stem cells in order to improve their regenerative potential [108]. Pre-conditioning with anoxic microenvironment increased the cardio-protective properties of MSCs in a model of diabetic cardiomyopathy [109]. Supplementing the culture media with a cocktail of potentiating cytokines could also be a viable option [110, 111]. However, it has to be taken into account that transplanted stem cells, either fortified autologous cells or healthy allogeneic cells, have to face a diabetic milieu which can obliterate any salutary improvement.

## Conclusions

In summary, BM cell therapy represents a potential opportunity for treatment of diabetic complications, yet its potential is not fully explored and realized. Even in the case in which this regenerative approach will prove to be marginally useful in a clinical setting, the concept of restoring the proper BM environment remains of vital importance and requires attention for tailoring novel therapies capable of restating global organ homeostasis in patients with DM.
